# Supplementary material for: Beyond Quasi-Particle Self-Consistent GW for Molecules with Vertex Corrections
Source: J Chem Theory Comput. 2025 Feb 11;21(4):1709–21. doi: 10.1021/acs.jctc.4c01639 (PMC11866760; doi:10.1021/acs.jctc.4c01639)
Supplement: Supplementary file 1 — ct4c01639_si_001.pdf [file ct4c01639_si_001.pdf]

Supporting information to "Beyond quasi-particle self-consistent GW for molecules with vertex corrections"

Arno Förster[1]

Theoretical Chemistry, Vrije Universiteit Amsterdam, De Boelelaan 1105,
1081 HV Amsterdam, The Netherlands

(*Electronic mail: a.t.l.foerster@vu.nl)

(Dated: 2 December 2024)

S1. CHARGED EXCITATION ENERGIES

TABLE S1. IPs, EAa, and and fundamental gaps calculated with qsΣ^{BSE}@L^{BSE} using aug-cc-pvDZ and aug-cc-pVTZ basis sets. All values are in eV,

	aug-cc-pvDZ			aug-cc-pvTZ		
**Compound**	**IP**	**EA**	**Gap**	**IP**	**EA**	**Gap**
Acridine	7.82	0.47	7.35	–	–	–
Anthracene	7.30	0.15	7.15	–	–	–
Azulene	7.40	0.24	7.16	7.60	0.46	7.14
Benzonitrile	9.78	−0.44	10.22	9.97	−0.28	10.25
Benzoquinone	10.39	1.34	9.05	10.54	1.50	9.03
Bodipy	8.01	1.40	6.61	–	–	–
Cl4-benzoquinone	10.05	2.27	7.79	10.21	2.43	7.79
Cl4-isobenzofuranedione	9.94	1.37	8.56	–	–	–
Dichlone	9.76	1.68	8.08	9.90	1.87	8.03
Dinitrobenzonitrile	11.06	1.54	9.52	11.24	1.74	9.50
F4-benzenedicarbonitrile	10.69	1.36	9.33	10.91	1.62	9.29
F4-benzoquinone	11.00	2.10	8.90	11.16	2.29	8.87
Fumaronitrile	11.12	0.78	10.34	11.34	1.00	10.34
Maleicanhydride	11.42	0.76	10.65	11.61	0.98	10.63
MDCNB	10.26	0.37	9.90	10.46	0.57	9.90
Naphthalenedione	9.85	1.26	8.59	9.98	1.40	8.58
NDCA	9.00	1.05	7.95	–	–	–
Nitrobenzene	10.13	0.29	9.85	10.31	0.48	9.83
Nitrobenzonitrile	10.52	1.12	9.39	10.69	1.31	9.39
Phenazine	8.25	0.88	7.37	8.37	1.02	7.35
Phthalicanhydride	10.50	0.60	9.90	10.65	0.78	9.87
Phthalimide	10.04	0.36	9.68	10.22	0.54	9.68
TCNE	11.66	2.89	8.78	11.89	3.14	8.75
TCNQ	9.29	3.30	5.99	–	–	–

TABLE S2. IPs, EAa, and and fundamental gaps calculated with qsGW@RPA using aug-cc-pvDZ and aug-cc-pVTZ basis sets. All values are in eV.

	aug-cc-pvDZ			aug-cc-pvTZ		
**Compound**	**IP**	**EA**	**Gap**	**IP**	**EA**	**Gap**
Acridine	7.81	0.59	7.22	8.06	0.85	7.21
Anthracene	7.30	0.25	7.05	7.52	0.49	7.03
Azulene	7.31	0.45	6.86	7.55	0.69	6.86
Benzonitrile	9.73	−0.38	10.11	9.96	−0.13	10.09
Benzoquinone	10.37	1.37	9.00	10.68	1.64	9.04
Bodipy	7.96	1.49	6.47	8.22	1.77	6.45
Cl4-Benzoquinone	10.07	2.34	7.73	10.28	2.55	7.73
Cl4-Isobenzofuranedione	9.88	1.49	8.39	10.14	1.76	8.36
Dichlone	9.73	1.76	7.97	9.95	1.98	7.97
Dinitrobenzonitrile	10.98	1.64	9.34	11.20	1.88	9.32
F4-Benzedicarbonitrile	10.59	1.48	9.11	10.83	1.77	9.06
F4-Benzoquinone	11.02	2.15	8.86	11.28	2.42	8.85
Fumaronitrile	11.23	0.76	10.47	11.46	1.03	10.43
MaleicAnhydride	11.38	0.78	10.61	11.70	1.06	10.65
mDCNB	10.22	0.44	9.78	10.47	0.71	9.76
Naphthalenedione	9.72	1.33	8.40	9.95	1.56	8.39
NDCA	8.94	1.13	7.81	9.20	1.40	7.80
Nitrobenzene	10.01	0.38	9.63	10.24	0.66	9.58
Nitrobenzonitrile	10.45	1.20	9.25	10.70	1.47	9.23
Phenazine	8.24	1.02	7.23	8.50	1.28	7.22
PhthalicAnhydride	10.36	0.69	9.67	10.59	0.95	9.64
Phthalimide	9.90	0.44	9.45	10.13	0.70	9.43
TCNE	11.69	2.88	8.81	12.00	3.20	8.80
TCNQ	9.34	3.28	6.06	9.59	3.59	6.00

TABLE S3. IPs, EAa, and and fundamental gaps calculated with qs$\Sigma^{BSE}@L^{TDH}$ using aug-cc-pvDZ and aug-cc-pVTZ basis sets. All values are in eV.

	aug-cc-pvDZ			aug-cc-pvTZ		
**Compound**	**IP**	**EA**	**Gap**	**IP**	**EA**	**Gap**
Acridine	7.62	1.09	6.54	–	–	–
Anthracene	7.15	0.76	6.39	–	–	–
Azulene	7.17	0.92	6.26	7.39	1.14	6.24
Benzonitrile	9.44	0.15	9.30	9.67	0.40	9.28
Benzoquinone	9.68	1.96	7.71	9.97	2.21	7.76
Bodipy	7.66	1.80	7.71	–	–	–
Cl4-benzoquinone	9.60	2.88	6.73	–	–	–
Cl4-isobenzofuranedione	9.51	1.94	7.56	–	–	–
Dichlone	9.33	2.25	7.07	–	–	–
Dinitrobenzonitrile	10.56	2.17	8.39	–	–	–
F4-benzenedicarbonitrile	10.15	1.94	8.21	10.46	2.30	8.16
F4-benzoquinone	10.49	2.61	7.88	10.82	2.97	7.85
Fumaronitrile	10.87	1.37	9.51	11.16	1.62	9.54
Maleicanhydride	10.78	1.25	9.53	11.11	1.50	9.61
MDCNB	9.94	0.98	8.96	10.20	1.26	8.93
Naphthalenedione	9.40	1.83	7.56	–	–	–
NDCA	8.69	1.62	7.07	–	–	–
Nitrobenzene	9.71	0.96	8.75	9.94	1.25	8.69
Nitrobenzonitrile	10.01	1.70	8.32	10.27	2.01	8.26
Phenazine	8.03	1.55	6.49	–	–	–
Phthalicanhydride	9.93	1.24	8.70	10.16	1.50	8.66
Phthalimide	9.59	0.97	8.63	9.82	1.25	8.57
TCNE	11.34	3.27	8.06	–	–	–
TCNQ	8.99	3.59	5.40	–	–	–

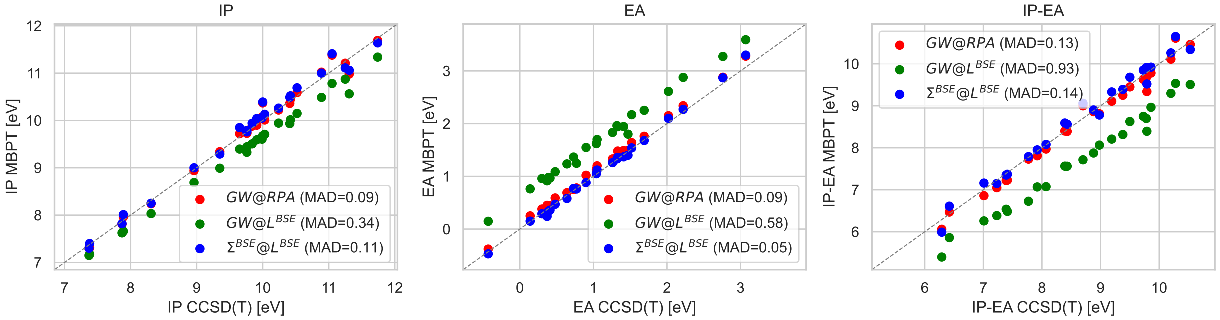

FIG. S1. Deviations of the molecules in the ACC24 set to ΔCCSD(T) for quasi-particle self-consistent GW@RPA, $GW@L^{BSE}$ and $\Sigma^{BSE}@L^{BSE}$ for IPs (left), EAs (middle) and fundamental gaps (right). All values are in eV. All values, including the reference values,[1] have been calculated using the aug-cc-pvDZ basis set.

S2. NEUTRAL EXCITATION ENERGIES

TABLE S4: BSE@qsGW and qsΣ^{BSE}@L^{BSE} neutral excitations for the QUEST#6 database.[2] The TBE (theoretical best estimate) values correspond to the ones calculated at cc-pVTZ.[2] All values are in eV.

			BSE@qsGW			qsΣ^{BSE}@L^{BSE}			
**Molecule**	**Transition**	**TBE**	**TZP**	**TZ2P**	**TZ3P**	**TZP**	**TZ2P**	**TZ3P**	**Primary contribution**
Aminobenzonitrile	$^1A_1$(CT)	5.26	5.08	5.08	5.07	5.31	5.31	–	4B2 → 5B2
Aniline	$^1A_1$(CT)	5.87	5.62	5.60	5.56	5.92	5.91	–	3b2 → 2a2, 1a2 → 4b2
Azulene	$^1A_1$(CT)	3.89	3.47	3.44	3.44	3.87	3.85	3.83	2a2 → 3a2, 3b2 → 4b2
	$^1B_2$(CT)	4.55	4.43	4.41	4.41	4.93	4.93	4.90	3b2 → 3a2
Benzonitrile	$^1A_2$(CT)	7.10	6.91	6.83	6.83	6.91	6.84	6.81	8B1 → 4B2
Benzothiadiazole	$^1B_2$(CT)	4.37	4.01	3.98	3.93	4.26	4.21	–	2a2 → 5b1
β-Dipeptide	$^1A'$(CT)	8.51	8.66	–	–	8.58	–	–	7a″ → 10a″, 7a″ → 9a″
	$^1A''$(CT)	8.90	8.89	–	–	8.65	–	–	31a′ → 9a″, 30a′ → 10a″
Dimethylaminobenzonitrile	$^1A_1$(CT)	4.94	4.85	4.85	–	5.03	5.03	–	5b2 → 6b2
Dimethylaniline	$^1B_2$(CT)	4.47	4.54	4.54	–	4.81	4.81	–	4B2 → 3A2
	$^1A_1$(CT)	5.54	5.39	5.38	–	5.65	5.65	–	4B2 → 5B2
Dipeptide	$^1A''$(CT)	8.15	8.53	–	–	8.43	–	–	28a′ → 8a″, 28a′ → 9a″
N-Phenylpyrrole	$^1B_2$(CT)	5.53	5.33	5.31	–	5.50	5.49	–	2a2 → 3a2
	$^1A_1$(CT)	6.04	6.09	6.08	–	6.31	6.28	–	2a2 → 5b2
Nitroaniline	$^1A_1$(CT)	4.57	4.37	4.34	4.31	4.64	4.63	–	4B2 → 5B2
Nitrodimethylaniline	$^1A_1$(CT)	4.28	4.13	4.10	–	4.35	4.34	–	5B2 → 6B2
Phthalazine	$^1A_2$(CT)	3.93	4.10	4.07	4.02	4.37	4.31	4.32	13B2 → 4B1
	$^1B_1$(CT)	4.34	4.46	4.42	4.37	4.87	4.81	4.79	13B2 → 3A2
Quinoxaline	$^1B_2$(CT)	4.74	4.37	4.36	4.32	4.65	4.65	4.62	–
	$^1A_1$(CT)	5.75	5.72	5.70	5.68	6.12	6.10	6.09	–
	$^1B_1$(CT)	6.33	6.10	6.07	6.05	6.49	6.47	6.44	–
Twisted DMABN	$^1A_2$(CT)	4.17	4.18	4.19	–	4.37	4.35	–	10B1 → 8B2
	$^1B_1$(CT)	4.84	4.97	4.97	–	5.16	5.15	–	10B1 → 3A2
Twisted PP	$^12B_2$(CT)	5.73	5.83	5.82	–	6.00	5.89	–	2A2 → 3A2
	$^12A_1$(CT)	5.82	6.04	6.03	–	6.25	6.01	–	9B1 → 9B2
Nitropyridine N-Oxide	$^12A_1$(CT)	4.24	3.94	3.93	3.92	4.36	4.39	4.30	4B2 → 5B2
Nitrobenzene	$^2A_1$	5.57	5.23	5.18	5.16	5.54	5.53	–	3b2 → 4b2

TABLE S5. Comparison of the lowest 5 excitation energies of Benzonitrile and Quinoxaline calculated with the cc-pVTZ and the TZ3P basis sets.

**Excitation**	**TZ2P**	**cc-pVTZ**	**Diff**
	**Benzonitrile**		
1	5.38	5.37	0.01
2	5.89	5.89	-0.01
3	6.81	6.79	0.02
4	6.96	6.97	-0.01
5	6.98	7.01	-0.03
	**Quinoxaline**		
1	4.07	4.05	-0.02
2	4.62	4.60	-0.02
3	4.69	4.66	-0.02
4	5.50	5.46	-0.03
5	5.98	5.98	0.00

REFERENCES

[1]R. M. Richard, M. S. Marshall, O. Dolgounitcheva, J. V. Ortiz, J. L. Brédas, N. Marom, and C. D. Sherrill, "Accurate Ionization Potentials and Electron Affinities of Acceptor Molecules I. Reference Data at the CCSD(T) Complete Basis Set Limit," J. Chem. Theory Comput. **12**, 595–604 (2016).

[2]P. F. Loos, M. Comin, X. Blase, and D. Jacquemin, "Reference Energies for Intramolecular Charge-Transfer Excitations," J. Chem. Theory Comput. **17**, 3666–3686 (2021), arXiv:2103.02947.
